# Potential Biomedical Application of Enzymatically Treated Alginate/Chitosan Hydrosols in Sponges—Biocompatible Scaffolds Inducing Chondrogenic Differentiation of Human Adipose Derived Multipotent Stromal Cells

**DOI:** 10.3390/polym8090320

**Published:** 2016-08-26

**Authors:** Anna Zimoch-Korzycka, Agnieszka Śmieszek, Andrzej Jarmoluk, Urszula Nowak, Krzysztof Marycz

**Affiliations:** 1 Department of Animal Products Technology and Quality Management, Faculty of Food Science, Wrocław University of Environmental and Life Sciences, 37 Chelmonskiego St., 51-630 Wrocław, Poland; anna.zimoch@up.wroc.pl (A.Z.-K.); andrzej.jarmoluk@up.wroc.pl (A.J.); 2 Department of Environment Hygiene and Animal Welfare, The Faculty of Biology and Animal Science, Wrocław University of Environmental and Life Sciences, 38 C Chelmonskiego St., 50-630 Wrocław, Poland; smieszek.agnieszka@gmail.com (A.Ś.); urszula.nowak.bio@gmail.com (U.N.); 3 Wroclaw Research Centre EIT+, Stablowicka 147, 54-066 Wroclaw, Poland

**Keywords:** sponges, adipose derived multipotent stromal cells, biocompatibility, chondrogenic potential, oxidative stress markers

## Abstract

Current regenerative strategies used for cartilage repair rely on biomaterial functionality as a scaffold for cells that may have potential in chondrogenic differentiation. The purpose of the research was to investigate the biocompatibility of enzymatically treated alginate/chitosan hydrosol sponges and their suitability to support chondrogenic differentiation of human adipose derived multipotent stromal cells (hASCs). The alginate/chitosan and enzyme/alginate/chitosan sponges were formed from hydrosols with various proportions and were used as a biomaterial in this study. Sponges were tested for porosity and wettability. The porosity of each sponge was higher than 80%. An equal dose of alginate and chitosan in the composition of sponges improved their swelling ability. It was found that equal concentrations of alginate and chitosan in hydrosols sponges assure high biocompatibility properties that may be further improved by enzymatic treatment. Importantly, the high biocompatibility of these biomaterials turned out to be crucial in the context of hydrosols’ pro-chondrogenic function. After exposure to the chondrogenic conditions, the hASCs in N/A/C and L/A/C sponges formed well developed nodules and revealed increased expression of collagen type II, aggrecan and decreased expression of collagen type I. Moreover, in these cultures, the reactive oxygen species level was lowered while superoxide dismutase activity increased. Based on the obtained results, we conclude that N/A/C and L/A/C sponges may have prospective application as hASCs carriers for cartilage repair.

## 1. Introduction

Nowadays, one of the fastest developing fields of medical science for humans and animals is regenerative medicine and tissue engineering. The emerging opportunity to regenerate damaged tissues and/or organs brings hope for treatment of many diseases involving the cardiovascular, endocrinological and the musculoskeletal systems [1,2,3]. Disorders of the locomotor system in general have become a serious medical problem, especially nowadays, when all over the world populations are rapidly aging. Moreover, the limitation of movement, chronic pain and disability present significant economic problems for global health care systems [4]. Bearing in mind the National Institute of Arthritis and Musculoskeletal and Skin Diseases reports, in USA alone more than 20 million citizens currently suffer from musculoskeletal system and related disorders including osteoarthritis (OA) [5]. These require medical solutions including the development of tissue engineered scaffolds combined with cellular therapies that together will recover lost tissue functions.

Articular cartilage, because of their low ability to self-renew, when injured, exhibits only limited regeneration capacity. The avascular character of cartilage impairs the regenerative process and leads to progressive tissue damage, losing integrity and finally functionality. The full recovery of its function, structure and biomechanical properties is hardly expected and, in most cases, results in degeneration [6]. To solve this issue, mesenchymal stromal stem cells, beside autologous chondrocyte implantation, have been proposed as a cellular candidate for cartilage regeneration [7]. Recently, the bone marrow (BMSCs) and adipose derived mesenchymal stem cells (ASCs) have been used more and more frequently in treatment of musculoskeletal system disorders—especially in the field of veterinary regenerative medicine [8]. In a wide range of animal model studies, including our own, the mesenchymal stem cells of adipose tissue (ASCs) have been successfully applied in the regeneration of cardiac tissues [9], neurological disorders and musculoskeletal tissues [10,11,12]. The MSCs exhibit great potential for self-renewal and ability to differentiate into multiple lineages [13,14,15]. Besides their proliferative and differentiation potential, their immunomodulatory and immunosuppressive properties make them a promising tool, especially in regenerative medicine of the musculoskeletal system [16]. The pro-regenerative potential of MSCs is explained by their paracrine activity and ability to secrete membrane derived vesicle (MVs), as well as their reach in a broad range of growth factors including transforming growth factor (TGF), bone morphogenetic protein type 2 (BMP-2), and vascular endothelial growth factor (VEGF), all crucial in the context of cartilage regeneration. Although the mechanism of MVs’ action is still poorly described, it seems that MVs, besides growth factors, may transfer anti-apoptotic and anti-inflammatory factors to the target cells [17,18,19]. Both human ASCs and BMSCs populations are characterized by similar cytophysiological features including surface marker character, in vitro expansion and long term culture stability. However, recently we showed that hASCs, in comparison to BMSCs, have greater potential for osteogenic differentiation when cultured onto metallic, and become an excellent model for testing the biocompatibility of biomaterials [20]. Bearing in mind these facts, in the present research we have used hASCs as an indicator of biocompatibility as well as cytotoxicity of tested polymers.

Rapid progress in the biomaterials science has resulted in the development of advanced 3D scaffolds, that might be promising regenerative solutions for different tissues/organ regeneration including articular cartilage [21,22]. Recent advances of regenerative medicine also focus on bioprinting technologies, in order to obtain precisely designed scaffolds for tissue repair and organ replacement. In these constructs, polymers (alginate) may be used as a bio-ink for chondrocytes’ encapsulation [23,24]. In comparison to metallic materials, the polymers have great advantage, because of their biocompatibility, biodegradability, and relatively low toxicity. Moreover, polymers for biomedical applications are well known from their reactivity, bioactivity, and water-binding capacity, as well as their ability to mimic the architecture of the native extracellular matrix (ECM). These seem to be crucial features that affect the behavior of transplanted cells including their attachment, viability as well as proliferative and differentiation potential. Both alginate and chitosan based biomaterials have been shown to be attractive for regenerative medicine as well as tissue engineering [25,26]. Indisputably, natural-based polymers offer an advantage over synthetic polymers with their similarity to ECM, what allows avoiding the stimulation of chronic inflammation or immunological reactions and toxicity [27]. The sodium alginate, a natural anionic polymer that is biodegradable and exhibits low cytotoxicity, has been shown to have a beneficial effect on cellular viability and tissue regeneration including cartilage and/or nerve tissue [28,29,30,31,32,33]. Our previous data indicate a moderately positive effect of sodium alginate on spinal cord regeneration in a lizard tail injuries model [34], that fully justifies the necessity for its further improvement. Additionally, 3D alginate scaffold, however, enriched in sphingolipids (S1P), has been shown to enhance the osteogenic differentiation potential of canine ASCs [35]. Unfortunately, pure sodium alginate based scaffold has limited ability to increase MSC viability, because of its low bioactive properties. In turn, chitosan, cationic, biodegradable, hemostatic and anti-bacterial polymers, which might be easily formed in 3D scaffold, possess interesting properties from a regenerative medicine perspective. Moreover, it was demonstrated that chitosan also has anti-oxidative properties that make it an even more promising biomaterial, and when it is applied in elderly patient, stem cells will be considered. Thus, combination of both alginate sodium and chitosan lead to obtaining polycation–polyanion (polyelectrolyte) complexes that might become an interesting hybrid biomaterial for cell based therapy. However, both alginate and chitosan are susceptible to various types of degradation including enzymatic, acid, alkaline, and/or oxidation-reduction that leads to glycosidic linkages and depolymerization. Recently, we have shown that 3D sponges obtained from alginate/chitosan substrate were characterized by elevated anti-oxidative properties that were strongly related with the percentage of added chitosan. Moreover, we have previously shown that addition of lysozyme significantly affects biomaterial morphology, rheology and solubility. In continuation of our recent study on the construction of sponges, we have focused on the physical properties, such as swelling ability and porosity, to fully characterize parameters which affect cell growth [36]. 

In the current research, we decided to test biocompatibility, including chondrogenic differentiation potential of human ASCs cultivated on previously characterized alginate/chitosan enzymatically treated scaffold. We additionally investigated the inflammatory activity of RAW264.7 in cultures with obtained biomaterials. In the current study, we used previously characterized biomaterials, choosing complexes that did not dissolve in the culture medium within 21 days of culture, since such time is required for the evaluation of MSCs’ chondrogenic differentiation potential. We have found that biomaterials with equal concentrations of chitosan and alginate as well as enzymatic treatment with lysozyme significantly enhanced hASCs viability, proliferative activity as well as chondrogenic differentiation potential. Moreover, we have found that alginate/chitosan 3D scaffolds that were enzymatically treated with lysozymes had reduced reactive oxygen species (ROS) levels and, at the same time, increased superoxide dismutase (SOD). These findings shed light on the prospect of alginate/chitosan 3D scaffolds enzymatically treated with lysozyme as candidates for ASCs as well as chondrogenic progenitor cells for biomedical applications.

## 2. Materials and Methods

### 2.1. Materials

All reagents used in this experiment were purchased from Sigma-Aldrich (Poznan, Poland), unless indicated otherwise.

All experimental procedures were approved by the II Local Ethics Committee of Environmental and Life Science University (Dec. No. 177/2010 of 15 December 2010) and the Local Bioethics Committee of Wroclaw Medical School (registry number KB-177/2014).

Low molecular weight chitosan (DD = 75%–85%; *M*_w_ = 150 × 103) and DL lactic acid (85% syrup) were obtained from Sigma Aldrich, Poznan, Poland. Alginate FD 901 AR was purchased from Danisco GRINDSTED^®^, Grindsted, Denmark. Lysozyme from white egg hen with 2000 U/mg activity was obtained from Ovopol, Nowa Sol, Poland.

### 2.2. Preparation of Enzyme/Alginate/Chitosan Hydrosols and Sponges

The preparation process has been previously described in our study [36]. Based on the results, four variants from the above mentioned study were chosen for testing. Chitosan (C) hydrosol was prepared in 1% aqueous solution of lactic acid by stirring at 400 rpm for 20 h. Alginate sodium (A) hydrosol was prepared in an analogous way. Enzyme: lysozyme (L) was dissolved in water by stirring at 300 rpm for 30 min and was added to A in activity of 1000 U. Then, both polymers (1%) were homogenized by homogenizer IKA (T18 basic, Ultra Turrax, Staufen, Germany) for 90 s in proportions shown in Table 1. Simultaneously, hydrosols were prepared also without lysozyme (N—no enzyme addition), as references. The mixtures were poured into polypropylene beakers of 60 mL and frozen at −80 °C for 24 h. Afterwards, the mixtures were lyophilized at −56 °C for 7 days.

### 2.3. Measurements of Porosity

The porosity of the AC sponges was calculated using liquid displacement method [37]. A known size and weight scaffold was immersed in a test tube containing preselected volume of ethanol (*V*_1_) in a vacuum closed chamber to evacuate air from the sponge’s pores. The volume of ethanol with sponge immersed in it was recorded after 1 h as *V*_2_. The volume of ethanol after sponge was removed was measured (*V*_3_). The void volume of the sponge was computed as *V*_1_–*V*_3_. The total volume of sponge was calculated from *V*_2_–*V*_3_ and the porosity of the sponge (ε) was evaluated as follows:

$$\varepsilon =\frac{V_{1}-V_{3}}{V_{2}-V_{3}}\times 100\%$$

### 2.4. Swelling Ability

The swelling ability of the AC sponges was determined by exposing the known weight (*W*_d_) to PBS solution for 30 min in room temperature as described Archana et al. [38]. The sponge was removed from the solution, blotted with filter paper and weighed. The water (*W*_sw_) absorbed by the sponge was calculated using the following equation:

$$W_{sw}=\frac{\left(W_{w}-W_{d}\right)}{W_{d}}\times 100\%$$

where *W*_w_ denotes the wet weight of scaffold immersed in the PBS and the distilled water after every 5 min.

### 2.5. Cell Populations

The analysis of biomaterials’ biocompatibility was performed using an in vitro model of human adipose tissue-derived multipotent stromal cells (hASCs) and monocyte-macrophage cell line i.e., RAW264.7 derived from ATCC (ATCC^®^ TIB-71^TM^, LGC Standards, Teddington, UK).

### 2.6. The Isolation and Propagation of hASCs

Isolation of human adipose-tissue derived mesenchymal stromal cells was performed as it was described previously [39]. All procedures were conducted under sterile conditions. Briefly, tissue biopsies were washed extensively with Hanks’s Balanced Salt Solution (HBSS) complemented with 1% antibiotic-antimycotic solution. Next, tissues were sliced into small pieces using surgical scissors. Tissue fragments were digested with 1 mg/mL collagenase type I for 40 min at 37 °C and then centrifuged for 10 min at 1200× *g*. Obtained pellets of stromal vascular fraction were suspended in Dulbecco’s Modified Eagle’s Medium (DMEM) with Nutrient F-12 Ham supplemented with 10% fetal bovine serum (FBS) and 1% antibiotic-antymycotic solution, and after that transferred to culture flasks. Primary cultures were maintained at 37 °C in an incubator with a humidified atmosphere of 5% CO_2_. When cultures reached approximately 80%, the passage of cells was performed. In order to obtain a sufficient number of cells for the experiment, cultures were passaged three times. Passage was performed using Accutase^®^ solution (Biowest S.A.S., Nuaillé, France) according to the manufacturer’s instructions. Following harvesting, cells were washed with HBSS at 300× *g* for 4 min.

### 2.7. Propagation of RAW264.7

Cultures of RAW264.7 were maintained at 37 °C in incubator with a humidified atmosphere. RAW264.7 were propagated in DMEM containing 4500 mg/L of glucose, supplemented with 10% FBS and 1% antibiotic-antymycotic solution. Medium was changed every two days. The passage was performed when cells reached approximately 80% confluence. For cells detachment the Accutase^®^ solution (Biowest S.A.S., Nuaillé, France) was used. The removal of enzyme and cells washing was performed at 125× *g* for 4 min. Cells used for the experiment were established at 10th passage.

### 2.8. Phenotypic Characterization of hASCs Using Flow Cytometry

Immunophenotyping of hACSs was investigated using fluorescence-activated cell sorting (FACS). The fluorochrome conjugated monoclonal antibodies were used for detection of CD34, CD44, CD45, CD73, CD90 and CD105 (all antibodies purchased from BD Pharmingen, San Jose, CA, USA). The antibodies used for analysis were preconjugated with specific fluorochrome: allophycocyanin (APC) for anti-CD73, peridinin chlorophyll protein complex (PerCP) for anti-CD105, fluorescein isothiocyanate (FITC) for anti-CD90, and phycoerythrin (PE) for anti-CD34, anti-CD44, anti-CD45. Preparation of cells for immunophenotyping proceeded as follows: ASCs were detached using Accutase^®^ solution, washed with HBSS supplemented with 2% FBS, and finally re-suspended at total of 5 × 10^5^ cells/mL. Next, cell suspensions were incubated for 20 min on ice with the specific antibodies (dilution 1:1000). Samples were analyzed on a Becton Dickinson FACS Calibur flow cytometer. Data analysis was performed using FlowJo software—trial version (TreeStar, Ashland, KY, USA).

### 2.9. Determination of Multipotent Properties of hASCs

Chondrogenic, osteogenic and adipogenic differentiation of hASCs was induced using commercially available kits (StemPro^®^, Thermo Fisher Scientific, Waltham, MA, USA) according to the manufacturer’s instructions. The cells were inoculated in 24-well plates at concentration of 2 × 10^4^ cells per well. Cultures maintained in non-differentiation medium i.e., complete growth medium suitable for hASCs culture served as control. Differentiation of cells was induced after 24 h of cells inoculation and was maintained for 21 days in case of osteogenic cultures, and 14 days for adipogenic and chondrogenic cultures. Growth culture media were replaced every three days.

Multilineage differentiation was confirmed using specific staining: Safranin-O for detection of glycosaminoglycans, Alizarin Red for calcium deposits and Oil Red O for neutral lipid deposits. Staining procedures were performed according to the manufacturer’s protocol. Differentiated cultures were analyzed using inverted microscope (Axio Observer A.1, Zeiss, Oberkochen, Germany), while the images were made using a PowerShot camera (Canon, Woodhatch, UK).

### 2.10. Proliferation Assay of hASCs and RAW264.7 in Cultures with Alginate/Chitosan Hydrosols in Sponges

Analysis of cells proliferation was performed in 24-well plates using transwell system with 8 µm pore polyethylene membranes (SPL Life Sciences Co., Ltd., Geumgang-ro, Korea). Biomaterials were placed in the upper chamber of the insert, while the cells were seeded in a density of 3 × 10^4^ (hASCs) or 6 × 10^4^ (RAW264.7) in the lower chamber. Both chambers were filled with 0.5 mL of complete growth medium. Each culture was performed in duplicate. The proliferation activity of cells was evaluated after 24, 72, and 98 h of culture. The results obtained for cultures with biomaterials were related to the cultures propagated on polystyrene. Proliferative activity of cells was monitored using resazurin-based assay (Alamar Blue test). The test was performed using protocol described in detail previously [37], in accordance with the manufacturer’s protocols. Proliferation factor (PF) was determined in relation to cell activity in cultures on polystyrene. A detailed description of the algorithm used for PF evaluation was presented in our previous paper [40]. Population doubling time was determined using the established method published by Jin et al. [41] with the use of an online calculator (http://www.doubling-time.com/compute.php, 2006, accessed 22 February 2016).

### 2.11. Measurement of DNA Synthesis in hASCs Cultures with Biomaterials

The synthesis of DNA in ASCs cultures with biomaterials was assessed by detection of 5-bromo-2-deoxyuridine (BrdU) incorporation test (Abcam, Cambridge, UK). The analysis was performed after 24, 72 and 96 h of the cells’ propagation in the presence of the biomaterials. Each culture was performed in triplicate. BrdU assay was performed according to the manufacturer’s protocol. Briefly, hASCs cultures were treated with BrdU reagent and incubated overnight at 37 °C in humidified atmosphere of 5% CO_2_ and 95% air. After that, cells were fixed and their DNA was denatured using a Fixing Solution for 30 min at room temperature. Next, anti-BrdU monoclonal antibody was added and cultures were incubated at room temperature for 1 h. Incubation with goat anti-mouse IgG conjugated with horseradish peroxidase (HRP) as secondary antibody was performed at room temperature for 30 min. Incubation with substrate—3,3′,5,5′-tetramethylbenzidine (TMB) was performed at room temperature in the dark for 30 min. Color reaction was stopped using solution provided by manufacturer. Intensity of signal was measured with a spectrophotometer microplate reader (Spectrostar Nano, BMG Labtech, Ortenberg, Germany) at a wavelength of 450/550 nm.

### 2.12. Examination of Morphology

Alterations in cells’ morphology were monitored intravitally after 24, 72 and 96 h of cultures with epifluorescent microscope (Axio Observer A.1, Zeiss, Oberkochen, Germany). For this purpose, cells were labeled with PKH67 Fluorescent Cell Linker Kit according to the manufacture’s information. Additionally, cultures propagated for 96 h were counterstained using diamidino-2-phenylindole (DAPI; 1:1000) and atto-565-labeled phalloidin (1:800). This microscopic observation required culture fixation. A detailed description of staining procedure was given previously [42]. Documentation of stained cultures was performed using a PowerShot Camera (Canon, Woodhatch, UK). Cultures’ morphology was also assessed using scanning electron microscope (SEM), accordingly to a well-established methodology described previously [42]. Cultures were analyzed using SE1 detector at 10 kV filament tension (SEM, Evo LS 15, Zeiss, Oberkochen, Germany) and 1000× magnification.

### 2.13. Analysis of hASCs Viability in Cultures with Biomaterials

The viability of hASCs in cultures with biomaterials was investigated using a two-color fluorescence live/dead assay (Double Staining Kit). The staining procedure was done according to the supplier’s recommendation. Analysis of cell viability was performed in cultures with biomaterials maintained in a transwell system. The experiment was conducted in duplicate. Cultures were observed under epifluorescence microscope (Axio Observer A.1) and captured using a PowerShot Camera. Obtained images were used for the evaluation of dead cell percentage. The calculations were performed using ImageJ software (National Institutes of Health, Bethesda, MD, USA, http://imagej.nih.gov/ij/).

### 2.14. Determination of Chondrogenic Character of hASCs

#### 2.14.1. Effectiveness of Chondrogenic Differentiation of hASCs on Alginate/Chitosan Hydrosols Sponges

For the test, cultures of hASCs were maintained in 24-well plates coated with biomaterials under chondrogenic and non-chondrogenic conditions. Cells were inoculated at a concentration of 3 × 10^4^ directly onto biomaterial. The experiment was performed in two independent replications. Chondrogenic differentiation of hASCs was induced at 24 h of hASCs propagation with specific commercial kit (StemPro^®^ Mesenchymal Chondrogenic Medium, Thermo Fisher Scientific). Growth media were changed every three days. After 14 days of culture, the chondrogenesis efficiency was analyzed.

#### 2.14.2. Detection of Chondrogenic Nodules

Chondrogenic differentiation of hASCs in cultures on biomaterials was assessed using proteoglycan-specific stain i.e., Alcian Blue. The procedure of staining included washing of cultures with HBSS and their fixation using 10% PFA. Cultures were maintained at 4 °C overnight. Subsequently cultures were rinsed twice with distilled water and stained with Alcian Staining Solution (5 g Alcian Blue in 50 mL of ethyl acetate). The staining was performed overnight at room temperature, in the dark. Following staining, cultures were incubated with ethyl acetate for 20 min at room temperature, in order to remove the unbounded dye. Finally, cells were washed with distilled water, until the stain was removed from preparations. Stained cultures were examined under inverted light microscope. Images were taken using a Canon PowerShot Camera.

#### 2.14.3. Analysis of Chondrogenic Nodules Formation and Extracellular Matrix Elemental Composition

The efficacy of chondrogenic nodules’ formation in biomaterials was assessed using high resolution scanning electron microscope combined with focused ion beam (FIB-SEM, Auriga Compact Crossbeam, Zeiss, Oberkochen, Germany). For observations, cultures within hydrosols were fixed overnight at 4°C in phosphate-buffered 2.5% glutaraldehyde (pH 7.3). After fixation, samples were washed three times in phosphate buffer for 5 min, and one time in deionized water for 3 min. Following washing, samples were dehydrated using the graded series of ethanol (from 50% to 100%, every 10%), each incubation lasting 5 min. Dry preparations were coated with gold particles using 300-s program (Scancoat Six, HHV Ltd., Crawley, UK). Prepared samples were imaged using field-emission scanning electron-ion cross-beam microscope (FIB-SEM). Morphometric analysis of chondrogenic nodules was performed with microscope software based on three different microphotographs. Distribution of molybdenum and potassium in chondrogenic nodules was determined using scanning electron microscopy combined with energy dispersive X-ray spectrometric analysis (EDS) (Evo LS 15, Zeiss, Oberkochen, Germany; Quantax, Bruker, Coventry, UK). Three samples from each cultures were measured and the results were shown as an average of repeats.

### 2.15. Analysis of Gene Expression: Real-Time Reverse Transcription Polymerase Chain Reaction (qRT-PCR)

After differentiation, chondrogenic cultures were homogenized using TRI Reagent. The *total*RNA was isolated accordingly to the method established by Chomczynski and Sacchi [43]. Obtained preparations were diluted in DEPC-treated water, and their quantity and purity was determined using spectrophotometer (WPA Biowave II, Biochrom Ltd., Cambridge, UK). Genomic DNA (gDNA) was removed from samples using DNase I RNase-free kit (Thermo Fisher Scientific, Waltham, MA, USA). Reverse transcriptase reactions were performed from 500 ng of *total*RNA using Tetro cDNA Synthesis Kit (Bioline Reagents Limited, London, UK). Both RNA purification and cDNA synthesis were performed on T100 Thermal Cycler (Bio-Rad, Hercules, CA, USA) in accordance with protocols supplied with kits. The quantitative PCR was performed using SensiFast SYBR & Fluorescein Kit (Bioline Reagents Limited, London, UK) in a total volume of 20 µL. The reaction was carried out on CFX Connect Real-Time PCR Detection System (Bio-Rad, Hercules, CA, USA). The cDNA did not exceed 10% of total volume of PCR. The concentration of primers in reaction was 500 nM. The detailed characteristics of primers are presented in Table 2. For the reaction, the following cycling conditions were applied: 95 °C for 2 min, followed by 60 cycles at 95 °C for 15 s, annealing for 15 s, and elongation at 72 °C for 15 s with a single fluorescence measurement. All reactions were performed in three repetitions. Specificity of the PCR products was determined by the analysis of the dissociation curve of amplicons. Melting curve was performed using gradient program of the range from 65 to 95 °C at a heating rate of 0.2 °C/s and with continuous measurement of the fluorescence. The average fold change in the gene expression of experimental cultures was compared with control cultures on polystyrene and calculated by the 2^−ΔΔ*C*t^ method in relation to the housekeeping gene [44].

### 2.16. Determination of Cytokines in Supernatants after RAW264.7 Cultures with Biomaterials

The concentration of cytokines i.e., TNF-α, p53 and IL-1 in supernatants collected after cultures were determined using specific enzyme-linked immunosorbent assays derived from R&D Systems (Minneapolis, MN, USA). Detection of IL-1 and total p53 was performed using DuoSet ELISA Development kit, with the sensitivity equal 50 ng/mL and 15.6 pg/mL, respectively. The TNF-α level was determined using Quantikine ELISA Kit with the sensitivity 7.21 pg/mL. For the analysis of IL-1 and p53, supernatants were undiluted, while determination of TNF-a required 4-fold dilutions. All tested samples and standards were measured in two replicates. Optical density was determined immediately after reactions and measured at 450 nm using a microplate reader (Spectrostar Nano, BMG Labtech, Ortenberg, Germany).

### 2.17. Oxidative Stress Factors in hASCs and RAW264.7 Culture

Reactive oxygen species (ROS) were measured using H2DCF-DA solution (Thermo Fisher Scientific, Waltham, MA, USA). Superoxide dismutase (SOD) was assessed using commercially available SOD determination kit. Both tests were performed in two replicates. The absorbance of the supernatants was measured spectrophotometrically at a wavelength of 560 nm and 450 nm, respectively, using a microplate reader (Spectrostar Nano, BMG Labtech, Ortenberg, Germany). ROS and SOD analysis was performed according to the manufacturer’s protocol. For ROS analysis, hASCs supernatants were undiluted, whereas supernatants of RAW264.7 required 2-fold dilution. For SOD analysis, all supernatants were undiluted. The ROS and SOD measurement in cultures with biomaterials was related to their activity in cultures on polystyrene.

### 2.18. Statistical Analysis

All experiments were triplicated. Data of porosity and swell ability of sponges were analyzed by one-way factor analysis of variance (ANOVA) with a significance level defined at *p* ≤ 0.05 using Statistica 9. The analysis of data obtained in biological assays were conducted with STATISTICA 10.0 software (StatSoft, Inc., Statistica for Windows, Tulsa, OK, USA). The normality of the population data was determined using Shapiro-Wilk test, while equality of variances was assessed by Levene’s test. Differences between groups were determined using one- or two-way analysis of variance (ANOVA). Differences with a probability of *p* < 0.05 were considered as significant.

## 3. Results and Discussion

### 3.1. Porosity

**Figure 1:**
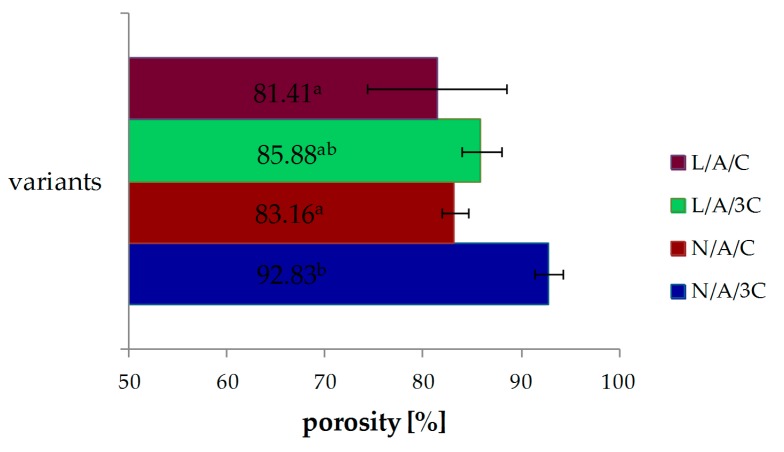
Effect of various sponges’ compositions on porosity, ^a–b^ values with different letters within the same column differ significantly (*p* < 0.05).

The porosity of CA sponges is shown in Figure 1. The porosity of the N/A/3C sponge was about 93%, while that of the L/A/3C sponge was about 86% and the difference between them is statistically significant. Similarly, it was observed that the porosity of N/A/C was about 83% and decreased after lysozyme addition to about 81%. With the increasing C content, the porosities of sponges were increased. The material porosity of above 80% was defined as ideal scaffold [37]. All reported porosities sponges were characterized with porosity of more than 80%. The nutrition and gases are exchanged in tissue engineering, when the scaffold has sufficient porosity [45]. Interconnection of pores assures proper cell migration, growth and proliferation and, therefore, facilitates tissue formation. That is why porosity is such an important factor in tissue engineering. The porosity of biomaterials is a crucial factor, with great relevance for scaffold-based tissue engineering strategies [46]. Next to the porosity, the pore size plays a crucial role in bone formation [47]. The pore sizes of N/A/C and L/A/C are similar and were 124.9 and 120 μm, respectively, as reported in a previous study. The largest pore size of 217.6 μm was obtained for L/A/3C [36]. The optimal pore size range in bone tissue engineering is 200–400 µm [48]. The optimal pore size range for skin tissues is 100–200 µm [49]. The optimal pore size range for cardiac tissues is 40–100 µm [50]. The pore size cannot be too small or too large, because the functions of the target tissues will not be maintained, causing reduction of viability [51]. However, the ideal range of pore size for in vitro chondrogenesis is still under evaluation and there are conflicting theories. Griffon et al. determine the effect of interconnective pore size (≤10 µm, 10–50 µm and 70–120 µm) on chondrocyte proliferation and function within chitosan sponges. They concluded that chondrocyte proliferation and metabolic activity were improved with increasing interconnective pore size of chitosan matrices [52]. However, pore size of gelatin scaffolds between 250 and 500 µm were preferable for better proliferation of chondrocytes [53]. It seems that ideal pore size strongly depends on used material in scaffold composition.

### 3.2. Swelling Ability

**Figure 2:**
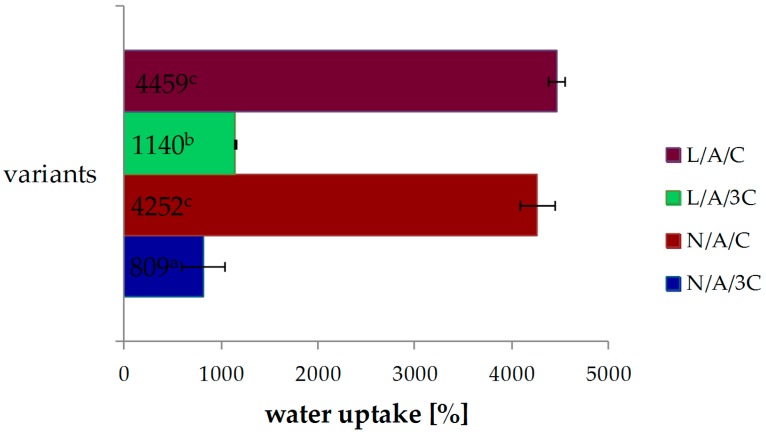
Effect of various sponges’ compositions on swelling ability; ^a–c^ values with different letters within the same column differ significantly (*p* < 0.05).

The water uptake ability of CA sponges is presented in Figure 2. The swelling ability of N/A/C sponges reached 4252% and was raised to 4459% in the sponge of L/A/C, but the difference was not significant. The water uptake of sponges decreased as the chitosan concentration increased. The N/A/3C sponge was found to have a swelling ability of 809%, while L/A/3C had one of 1140%. The lysozyme addition improved the swelling ability of sponges.

Swelling ability is one of the factors which determines the practical use of scaffold in tissue engineering. It helps in absorbing culture media, enhancing cell permeation in the scaffold and improving the internal surface area of the scaffold [38]. The alginate addition increased the swellability of scaffolds in our study as well as the composition of chitosan-alginate and chitosan-pectin-alginate reported by Archana et al. [38]. They have observed that an increase of swelling ability causes a decrease of the mechanical properties of scaffold, what was also observed in our previous study [36].

### 3.3. Characterization of Cells Used in the Experiment

**Figure 3:**
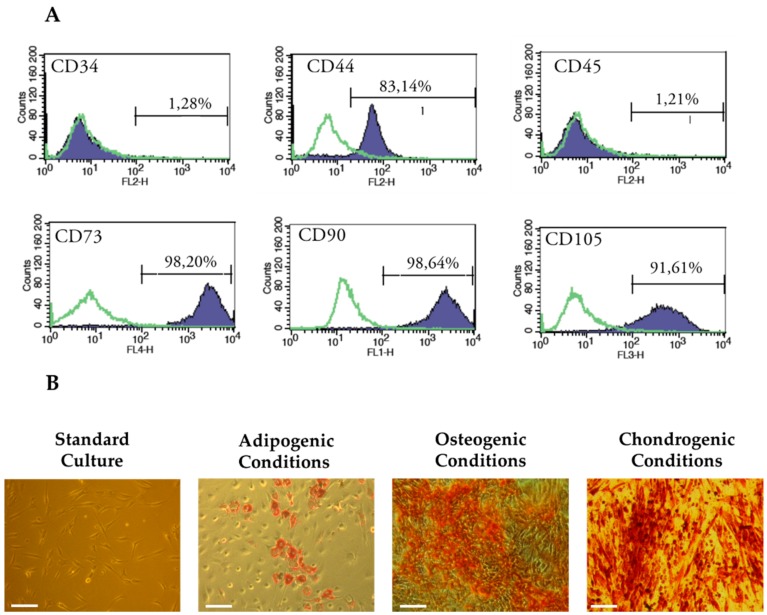
The results of immunophenotyping and multipotency assay of population of human adipose-derived stem cells used in the experiment. Flow cytometry revealing the positive expression of CD44, CD73, CD90 and CD105 mesenchymal markers and negative expression of CD34 and CD45 (**A**); The morphology of hASCs in standard, adipogenic, osteogenic and chondrogenic culture was presented (**B**), scale bar = 100 µm. The lipid-rich vacuoles in adipogenic cultures were stained with Oil Red O, the calcium deposits in osteogenic cultures were detected using Alizarin Red, while accumulation of proteoglycans in chondrogenic cultures visualized using Safranin-O.

The human ASCs used in this experiment fulfilled the criteria established by International Society of Cellular Therapy [54]. We obtained adherent fibroblast-like cells, expressing specific surface markers (CD44, CD73, CD90, and CD105) and lacking expression of CD34 and CD45 i.e., markers of haematopoietic origin. The characteristics of hASCs immunophenotype are presented in Figure 3A. Additionally, obtained hASCs exhibited potential to differentiate not only into cells of adipose tissue origin, but also into bone and cartilage cells i.e., osteoblast and chondrocytes, respectively. Formation of tissue-specific extracellular matrix was confirmed with specific staining, what is shown in Figure 3B.

### 3.4. Results of Biocompatibility Assessment

#### 3.4.1. Proliferation of Cells in Cultures with AC Sponges

**Figure 4:**
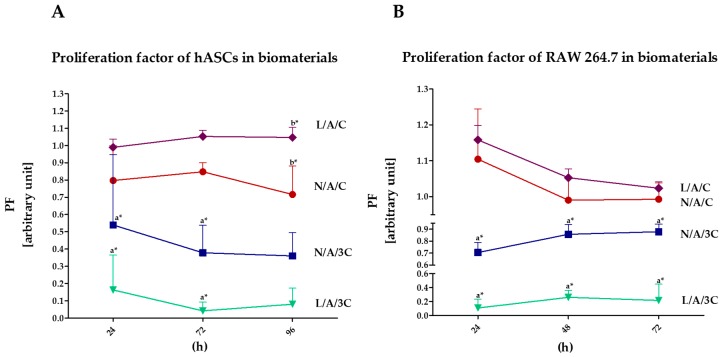
Proliferation activity of cells: (**A**) ASC; (**B**) RAW264.7 in biomaterials. The *X*-axis refers to the time of cells’ propagation. An asterisk (*) marks a statistically significant difference (*p* < 0.05).

**Figure 5:**
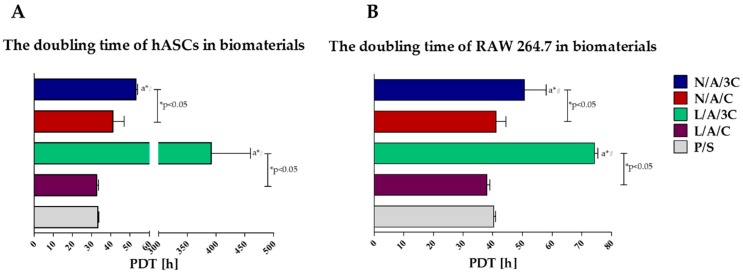
The doubling time of cells: (**A**) ASC; (**B**) RAW264.7. An asterisk (*) indicates a statistically significant difference (*p* < 0.05) between biomaterials, while hashtag (#) in comparison to cultures on polystyrene (P/S).

The highest proliferation activity, both of hASCs and RAW264.7, was noted in cultures with L/A/C sponge, while the lowest in cultures with L/A/3C sponge (Figure 4). The kinetic growth of hASCs on L/A/C sponge had an exponential nature, while the intense proliferation of RAW264.7 was associated with a decline of cells’ proliferative activity. Obtained results indicate that lysozyme addition improves cell proliferation in cultures with sponges characterized by equal AC ratio. The increase of chitosan concentration in sponges had a negative effect on cell proliferation, moreover, the enzymatic treatment of N/A/C sponges with lysozyme did not enhance proliferative activity of hASCs, but significantly reduced it. Analysis of population doubling time of hASCs and RAW264.7 in cultures with AC sponges confirmed that lysozyme addition has a beneficial effect on cell proliferation only when the AC ratio in sponges is established to be 1:1, while the sponges with increased chitosan concentration significantly retards the population doubling time of cultures. Moreover, the enzymatic treatment of N/A/3C notably prolongs doubling time, both of hASCs and RAW264.7 (Figure 5). The positive influence of alginate–chitosan complexes on cell proliferation was noted previously. The beneficial effect of chitosan addition on cell attachment was shown, thus cell proliferation was emphasized. It was shown that alginate–chitosan scaffolds are characterized by greater cell adhesion compared to alginate-alone [55]. Interestingly, our results indicate that increase of chitosan concentration ratio in AC sponges negatively affects cells’ proliferative activity. Considering the role of high swelling ability of N/A/C and L/A/C, it seems that this characteristic, along with high porosity may improve cell proliferation. Obtained results are in good agreement with previous studies showing that high water swelling ability may improve during the wound healing process, strongly dependent on the proliferation of cells. Bearing in mind the distinguished porosity of N/A/3C and L/A/3C, we expected that cell proliferation in cultures on these sponges will be improved; in turn, we noted the lowered cell activity both in the case of hASCs and in RAW264.7 cultures. The data obtained here is contrary to studies by Ji et al. [56] that showed that increased porosity positively influences cell proliferation by enhancing nutrients and waste exchange. This may be due to the fact that chitosan at high concentrations may exert a cytotoxic effect. The cytotoxicity of chitosan materials depends on its concentration and molecular weight [57]. Another study has also proved that other physical properties of chitosan may influence its biological properties. This includes the depolymerization degree as well as zeta potential [58].

#### 3.4.2. The Influence of AC Sponges on Cells Morphology and Growth Pattern

**Figure 6:**
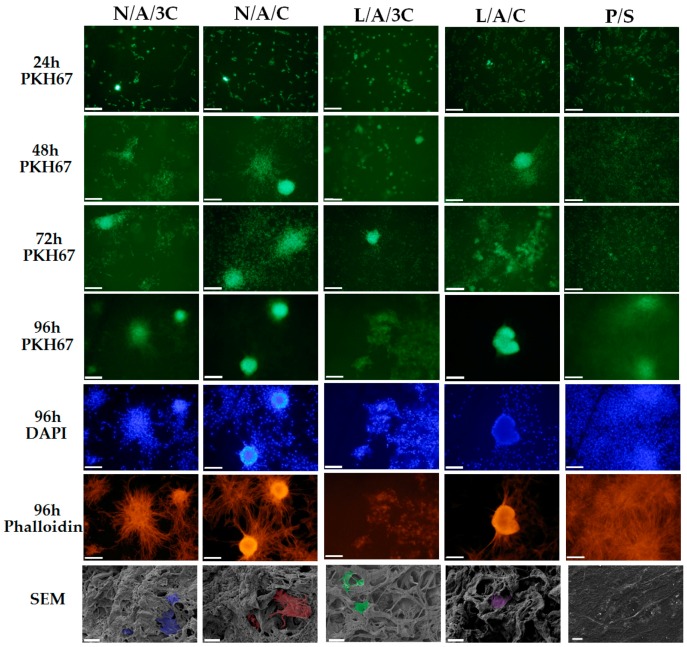
The morphological changes of hASCs after 24, 48, 72 and 96 h in the cultures. The cells labeled with PKH67 (light green membranes) were monitored during the experiment. Cultures at 96 h were also stained with atto-565 phalloidin for the visualization of cytoskeleton (red stained cell bodies) and with DAPI for nuclei (blue dots), Magnification used is 100×, and scale bar is 200 μm. The cultures at 96 h were observed using SEM. Images were captured at magnification 1000-fold, scale bar = 40 and 20 μm.

**Figure 7:**
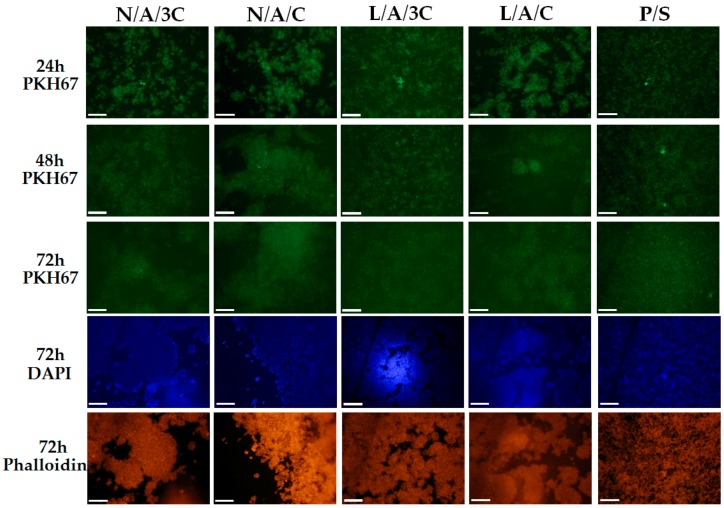
The morphological changes of RAWs264.7 after 24, 48 and 72 h in the cultures. Cellular membranes were stained with PKH67 (light green), nuclei were visualized with DAPI stating (blue dots), while actin cytoskeleton was visualized using atto-565 phalloidin (red stained cell bodies). Magnification used is 100×, and scale bar is 200 μm.

**Figure 8:**
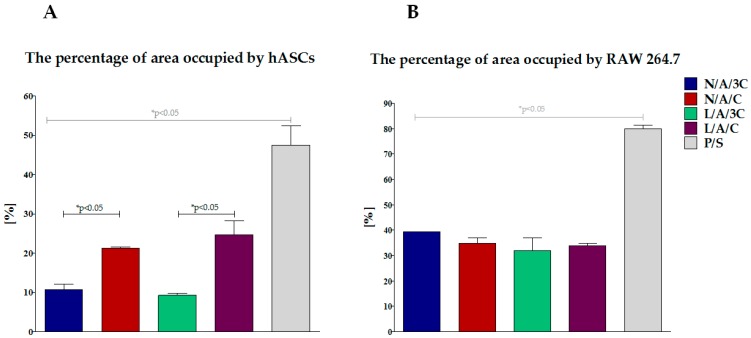
The percentage of area occupied by cells: (**A**) ASC; (**B**) RAW264.7. An asterisk (*) indicates a statistically significant difference (*p* < 0.05).

Morphology of human ASC cultured onto investigated sponges was monitored during 98 h of normal, non-chondrogenic cultures. Interestingly, we have observed that cells cultivated onto investigated sponges beside L/A/3C material exhibited spherical morphology that is typical for chondrogenic precursors’ cell shape (chondrogenic aggregates). We have found that after 98 h of culture, the biggest and most abundant chondrogenic like aggregates were observed in L/A/C and N/A/C sponges (Figure 6). The observed cellular aggregates were characterized by a well-developed cytoskeleton when compared to the control, polystyrene 2D culture (P/S). The observed morphological changes of hASC cultivated onto L/A/C and N/A/C sponges might indicate a pro-chondrogenic character of L/A/C and N/A/C biomaterials. These clearly demonstrate that 3D sponges in particular and L/A/C and N/A/C promote creation of chondrogenic-like nodules, and induce normal hASC chondrogenic morphology. Similarly to our observation, recently Song and colleagues have observed that three-dimensional alginate based sponge induces chondrocytes’ formation in vitro [59]. In addition, they found that alginate based sponge effectively minimalizes inflammatory reaction through reducing the content of mannuronic acid causing immune rejection. These findings stand in partially good agreement with our observation, where both L/A/C and N/A/C sponges significantly reduced the space occupied by RAWs264.7 (Figure 7 and Figure 8B). Moreover, we observed that N/A/C and L/A/C sponges respectively affect the hASC growth pattern and surface occupation. We have found that, among tested sponges, the hASC grew most expansively onto N/A/C and L/A/C materials (Figure 8A). This indicates that both tested biomaterials had a beneficial effect on cellular growth and are attractive for material expansion when compared to the other tested sponges.

#### 3.4.3. Cytotoxicity of AC Sponges

**Figure 9:**
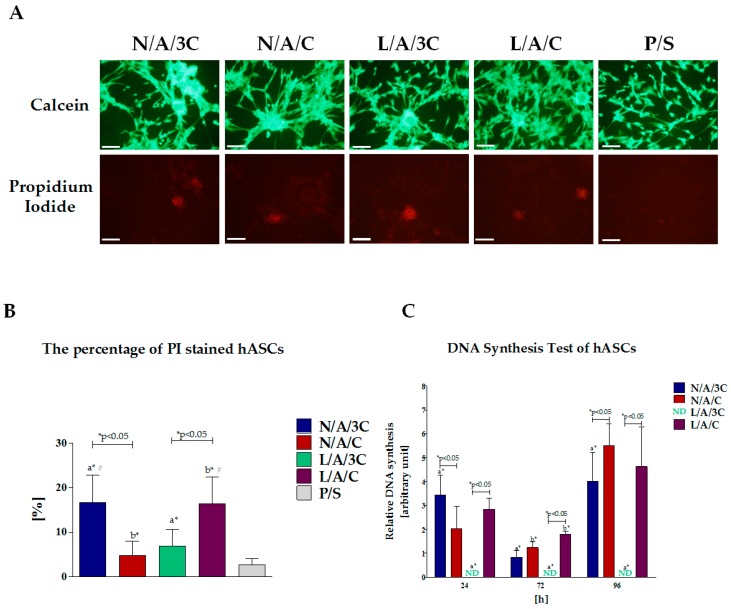
Cytotoxicity of biomaterials and influence of DNA synthesis in hASC cultures. Images of cells stained with calcein and propidium iodide (**A**), scale bar =200 μm; Comparative analysis of percentage of propidium iodide positive cells (**B**); Results of BrdU incorporation assay (**C**). Fold change in DNA synthesis was calculated by comparing BrdU signals of hASCs maintained with biomaterials to that of the control culture, which was assigned a value of 1. An asterisk (*) indicates a statistically significant difference (*p* < 0.05) for comparative analysis of changes related to the biomaterial composition, while hashtag (#) indicates differences between cultures on biomaterials and polystyrene.

To determine whether the AC sponges may influence the apoptosis level of hASCs cultures, we analyzed the percentage of dead cells using a calcein and propidium iodide (Figure 9A) double staining procedure. Obtained results showed that apoptosis involved cells that formed aggregates in cultures with biomaterials. The quantification analysis of the image data (Figure 9B) showed a significant increase of dead cells in cultures with N/A/3C and L/A/C sponges. The lowest percentage of dead cells was noted in hASCs cultures with N/A/C. Enzymatic treatment of sponges with increased chitosan concentration caused reduction of death cell percentage. In case of cultures with L/A/C sponges, we noted an increased number of propidium iodide-positive cells. To determine the accurate state of hASCs proliferation and to assess the relationship of biomaterial cytotoxicity with the level of DNA synthesis, we performed analysis of BrdU incorporation during the S-phase of the cell cycle. Obtained data (Figure 9C) are consistent with results from the cell proliferation activity assay (Figure 4A). The highest number of hASCs during S-phase was noted in cultures on N/A/C and L/A/C sponges. The significantly higher synthesis of DNA under the influence of L/A/C was noted at 48 h of hASCs culture. However, it seems that during the adaptive and early growth phase, as well as during stationary phase of cultures, the modification of N/A/C sponges by lysozyme addition had no significant influence on DNA synthesis level. The increased chitosan concentration in N/A/3C sponge positively influenced the cell cycle progression and DNA replication only at 24 h of the hASC culture. The signal derived from cells entering the cell cycle during S-phase was not detectable in cultures with L/A/3C. Obtained data correlates with proliferative activity of hASCs in cultures with L/A/3C which additionally indicates the cytotoxic character of this biomaterial.

#### 3.4.4. Biomaterial-Induced Cytokine Expression in RAW264.7 Monocyte-Macrophage Cell Line

**Figure 10:**
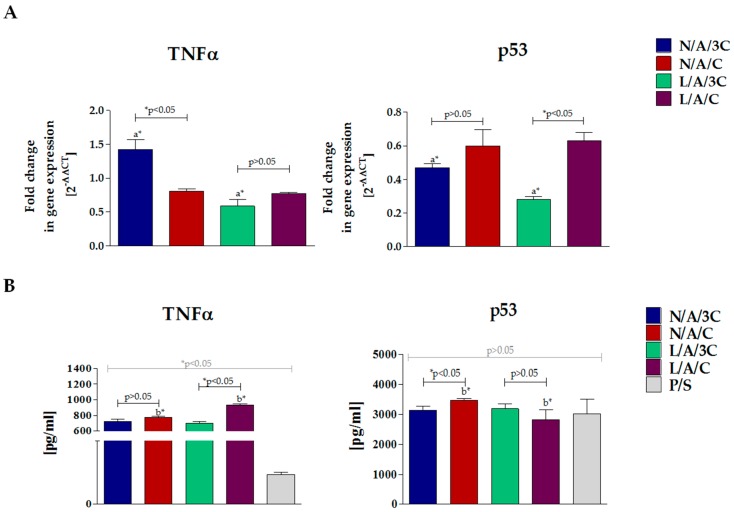
(**A**) Results of analysis of cytokine expression in RAW264.7 cultures with biomaterials. Gene expression of *p53* and *TNF*-*α* was determined with qPCR, 2^−ΔΔ*C*t^ method was used to calculate the quantification of relative values, normalizing data to control and including expression of reference gene (*ACTβ*). The secretion of cytokines measured with specific ELISA test (**B**). Error bars represent standard deviation from the mean calculated for normalized values obtained in three separate replicates. An asterisks (*) represent statistically significant differences (*p* ˂ 0.05).

The expression of TNF-α and p53 in RAW264.7 cultured with AC sponges was determined both on mRNA as well as protein level (Figure 10). The highest expression of p53 was noted in cultures with N/A/C—in this culture, the high level of *p53* transcripts was correlated with p53 production. This dependency was not observed in RAW264.7 cultures on L/A/C—despite high level of mRNA for *p53*, we did not observe increased p53 protein secretion. The comparative analysis of relative fold expression showed that the overall *p53* transcript level was lower than in cultures on polystyrene; moreover, the secretion of p53 in cultures with biomaterials was comparable with p53 concentration in supernatants derived from cultures on polystyrene. The results of p53 expression in macrophages along with the results of propidium iodide test on hASCs (Figure 9) indicate that obtained biomaterials exert low pro-apoptotic effect on cells.

The transcript level of *TNF*-*α* in RAW264.7 cultures on N/A/C and enzymatically treated hydrosol sponges was lower than in cultures on polystyrene. However, in all RAW264.7 3D cultures, we noted enhanced TNF-α secretion. The increased production of TNF-α could be associated with intensive proliferation of RAW264.7 in cultures with biomaterials. Additionally, the alginate component may induce autocrine secretion of TNF-α in RAW264.7 cells crucial for not only macrophage proliferation but also the differentiation process [60,61]. 

#### 3.4.5. Assays of Oxidative Stress Factors in hASCs and RAW264.7 Cultures with Biomaterials

**Figure 11:**
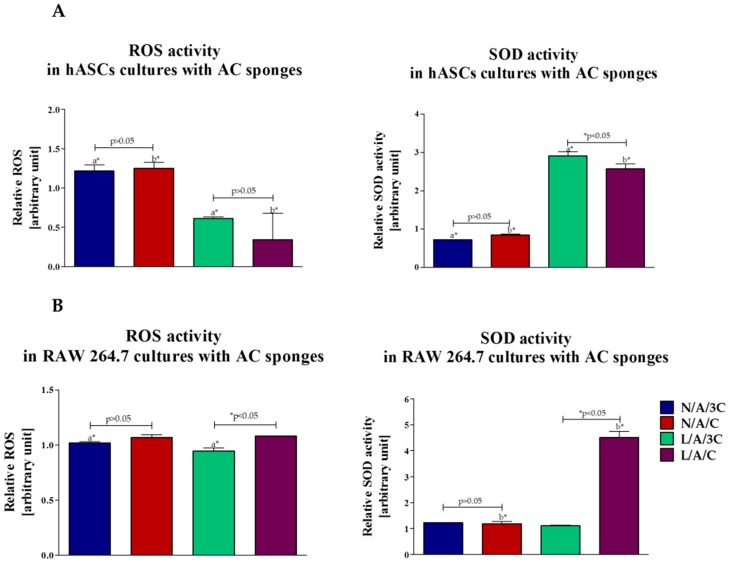
Analysis of oxidative stress factor in hASCs (**A**) and RAW264.7 (**B**): reactive oxygen species (ROS) and superoxide dismutase (SOD). An asterisk (*) represents statistically significant differences (*p* ˂ 0.05).

Analysis of oxidative stress factors in hASC cultured in tested sponges was performed at the last stage of non-chondrogenic culture. Reactive oxygen species (ROS) level significantly increased in N/A/3C and N/A/C sponges, while in L/A/3C and L/A/C sponges the lowest ROS level was observed. Interestingly, the lowest level of ROS in L/A/C sponges was accompanied by the highest concentration of superoxide dismutase (SOD) activity (Figure 11A). Obtained data strongly correlates with the elevated proliferative activity and viability of hASCs cultured onto L/A/C sponges. The increased SOD activity may also contribute to the low percentage of dead cells in L/A/3C sponges. Our previous studies indicate that oxidative stress factors, including reactive oxygen species (ROS) and nitric oxide (NO), may negatively impact hASC viability, proliferation, and differentiation. Disruption of the balance between free radicals and antioxidants may also influence the multipotent character of ASCs, and thus the phenotypic plasticity of cells resulting in lower ability for differentiation toward chondrocytes [62,63,64]. Obtained data indicating elevated anti-oxidative properties of enzymatically treated sponges stand in good agreement with our other findings, where we have shown that enzymatically treated A/C sponges have higher anti-oxidative properties when compared to sponges without lysozyme addition. Moreover, we have observed an elevated SOD activity in RAW264.7 that was cultured onto L/A/C sponges (Figure 11B). This sheds light on its potential in enzymatically-treated sponges in the context of its anti-oxidative features for potential biomedical application.

### 3.5. Chondrogenic Differentiation of hASCs in AC Sponges

**Figure 12:**
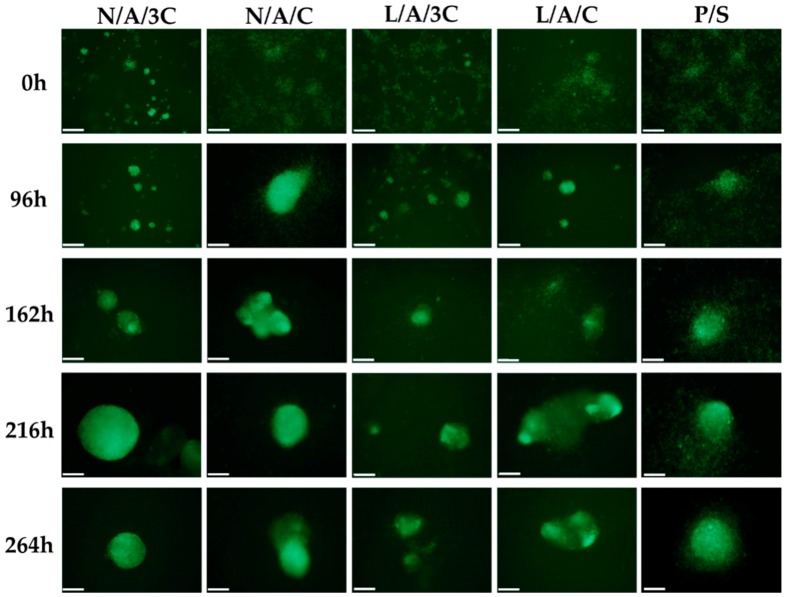
Visualization of nodules development during chondrogenic cultures of hASCs. The morphological changes were monitored during chondrogenesis (from 0 to 264 h of chondrogenic induction). Cell membranes were stained with PKH67 (light green). Magnification used for analysis was 100×. Scale bar equals 200 μm.

The high biocompatibility of N/A/C and L/A/C sponges reflected on their distinguished chondroinductive properties. The hASCs labeled with PKH67 were monitored during the course of chondrogenesis; therefore, we were able to determine the development of cellular aggregates within the investigated AC sponges (Figure 12).

**Figure 13:**
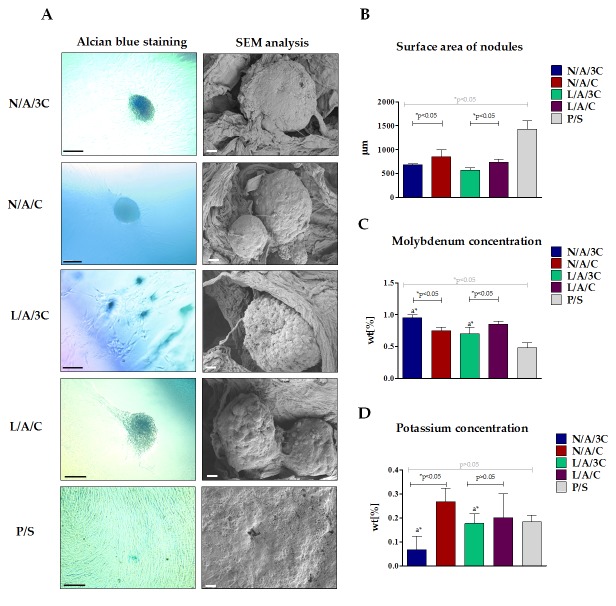
Characterization of chondrogenic cultures of hASC in 3D (AC sponges) and 2D systems (polystyrene). The Alcian Blue staining and SEM analysis of chondrogenic nodules; scale bar = 100 μm and for microphotographs = 2 μm (**A**). The results of surface area of nodules determination (**B**). Analysis of chondrogenic nodules elemental composition in relation to molybdenum (**C**) and potassium (**D**) concentration. An asterisk (*) indicates a statistically significant difference (*p* < 0.05).

The sponges’ porosity influenced spontaneous cell aggregation and, therefore, promoted formation of proteoglycan-rich chondrogenic nodules that were visualized by Alcian blue staining (Figure 13A). In turn, the reaction with Alcian Blue, in chondrogenic cultures propagated on polystyrene was weak. The morphometric analysis of nodules (Figure 13B) showed that the most developed chondrogenic aggregates were formed in cultures in N/A/C, however, nodules formed in sponges enzymatically treated diminished in size. Despite the fact that cellular aggregates in cultures on polystyrene occupied more surface area, the typical cartilaginous nodules were not developed. Obtained results indicate that 3D structure of AC sponges facilitates cell–cell contact and promote formation and functional development of nodules in short-term chondrogenic cultures, when compared to the monolayer expansion on polystyrene. This observation is in good agreement with previous studies showing that 3D porous alginate-chitosan scaffolds provide a favorable microenvironment for supporting proliferation and chondrogenic differentiation of progenitor cells [65,66].

The elemental composition of chondrogenic aggregates formed in cultures on AC sponges showed accumulation of molybdenum and potassium (Figure 13C,D). Both molybdenum and potassium are chemical elements of particular importance for cartilage tissue development—molybdenum as a cofactor for enzymes is involved in the reconstruction of the cartilage, whereas potassium channels are crucial for chondrocyte biosynthetic activity [67,68]. The highest concentration of molybdenum was noted in chondrogenic cultures’ nodules formed by of hASCs on N/A/3C. In all other cultures, the molybdenum concentration in nodules was comparable. Nevertheless, the concentration of molybdenum in nodules formed in cultures within AC sponges was significantly higher than in cellular aggregates formed in chondrogenic cultures on polystyrene. The potassium accumulation in nodules decreased with the increased chitosan concentration in sponges. The lysozyme treatment of AC sponges in the case of a group with a 1:3 ratio resulted in formation of nodules significantly richer in potassium, while in the case of AC sponges characterized by 1:1 ratio the enzymatic treatment caused a decrease in potassium level, however, not statistically significantly. Moreover, the comparison of potassium level between cultures on AC sponges and on biomaterial did not show statistically significant differences.

**Figure 14:**
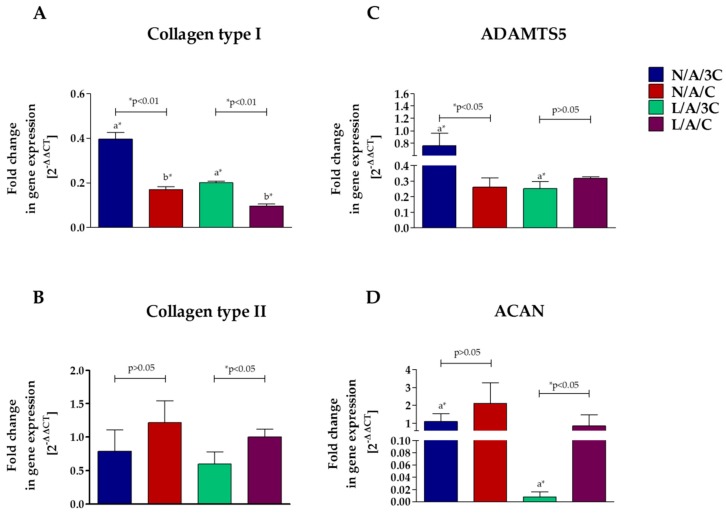
The results of gene expression analysis of *Coll-1* (**A**); *Coll-2* (**B**); *ADAMTS5* (**C**) and *ACAN* (**D**) in hASCs cultured on biomaterials. The 2^−ΔΔ*C*t^ method was used to calculate the quantification of relative values, normalizing data to control and including expression of reference gene (*GAPDH*). Error bars represent standard deviation from the mean calculated for normalized values obtained in three separate replicates. An asterisk (*) represents statistically significant differences (*p* ˂ 0.05).

The analysis of gene expression of chondrogenic markers (Figure 14) showed that cultures propagated within N/A/C sponges were characterized by increased expression of cartilage specific markers i.e., *Coll-2* and *ACAN* and lowered expression of *Coll-1* and *ADAMTS5*. Interestingly, we noted the decrease in expression of collagen type I and *ADAMTS5* in chondrogenic cultures in sponges L/A/3C, however, this was also associated with a decrease of mRNA. The lysozyme treatment of A/C biomaterials did not influence the expression of *Coll-2* and *ACAN*, but it influenced the lowered expression of *Coll-1*. Interestingly, we also noted the decrease in expression of collagen type I and *ADAMTS5* in chondrogenic cultures in sponges L/A/3C, however, this was also associated with decrease of mRNA for *ACAN*.

Obtained results indicate that the most effective chondrogenic differentiation of hASCs was seen in 3D cultures on N/A/C and L/A/C. The functional acceleration of chondrogenesis was reflected with the formation of well-developed nodules rich in molybdenum and potassium but also with the expression profile of cartilage-specific genes. In chondrogenic cultures of hASC on N/A/C and L/A/C, the expression of ACAN and Coll-2 was associated with a decrease of Coll-1 and aggrecanase, which might be correlated with the formation of proper, functional cartilage microtissue. Our results are in good agreement with the previous observations that porous AC scaffolds may induce chondrogenic differentiation of cells reflected by significant synthesis and accumulation of ECM macromolecules [69]. The polymer blend's porosity is a crucial feature that promotes formation of cellular clusters and, therefore, naturally enhances progenitor cells’ differentiation toward cartilage producing cells. 

## 4. Conclusions

Obtained results indicate that biomaterials distinguished by high swelling ability, composed of alginate and chitosan in equal proportions, are biocompatible and non-toxic, both in cultures with human progenitor cells of mesenchymal origin, as well as in cultures with mouse macrophage cell line RAW264.7. Additionally, the porosity of obtained sponges promoted in non-differentiating cells a chondrogenic morphotype. Moreover, N/A/C and L/A/C sponges strongly affected the chondrogenic potential of hASCs, which was manifested by the formation of functional chondrogenic nodules, as well as a characteristic transcriptome profile associated with increased expression of collagen type II, aggrecan and decreased expression of collagen type I. Moreover, the progenitor cells in cultures with enzymatically treated sponges manifested low ROS levels and elevated SOD activity. Despite the fact that these results are not strictly related with enhanced chondrogenic differentiation of progenitor cells, we think that lysozyme treated polymers may support chondrogenesis potential by reducing oxidative damage to cells or tissues, thus they may find application in cartilage repair and osteoarthritis therapy, especially in elderly people.

The results prompt us to state that developed AC sponges may find application in regenerative medicine to improve cartilage repair and regeneration.

## Figures and Tables

**Table 1:** Enzyme/alginate/chitosan hydrosols composition.

Enzyme addition (E)	Polymers ratio A:C	Variants (E/A/C)
(U)	(*v*/*v*)	(Code)
N *	1:3	N/A/3C
	1:1	N/A/C
1,000 L	1:3	L/A/3C
	1:1	L/A/C

* No enzyme addition; A: sodium Alginate; C: Chitosan.

**Table 2:** Sequences of primers used in qRT-PCR.

Gene	GenBank accession number	Primer sequence	Annealing temperature (°C)	Amplicon size (bp)
**Human transcripts**				
*ACAN*	NM_001113455.2	F: GAGCCTGAAAACCAGACGGA	570	109
		R: TCTCCTCTGTTGCTGTGCTG		
*ADAMTS5*	NM_007038.3	F: TATGACAAGTGCGGAGTATG	60.8	182
		R: TTCAGGGCTAAATAGGCAGT		
*Coll-1*	NM_001003090.1	F: ACCGACCAAGAAACCACAGG	61.1	226
		R: GCACGGAGATTCCTCCAGTT		
*Coll-2*	NM_001006951.1	F: GACAATCTGGCTCCCAAC	60.3	233
		R: ACAGTCTTGCCCCACTTAC		
*GAPDH*	NM_001003142.2	F: GATTGTCAGCAATGCCTCCT	58.0	198
		R: GTGGAAGCAGGGATGATGTT		
**Mouse transcripts**				
*ACTβ*	NM_007393.5	F: CGACGATGCTCCCCGGGCTGTA	62.0	574
		R: CTCTTTGATGTCACGCACGATTTCCCTCT		
*p53*	NM_011640.3	F: AGTCACAGCACATGACGGAGG	61.0	287
		R: GGAGTCTTCCAGTGTGATGATGG		
*TNFα*	NM_013693.3	F: ACAGAAAGCATGATCCGCGA	62.0	295
		R: CTTGGTGGTTTGCTACGACG		
